# Digital Absolute Gene Expression Analysis of Essential Starch-Related Genes in a Radiation Developed *Amaranthus cruentus* L. Variety in Comparison with Real-Time PCR

**DOI:** 10.3390/plants9080966

**Published:** 2020-07-30

**Authors:** Veronika Lancíková, Andrea Hricová

**Affiliations:** Institute of Plant Genetics and Biotechnology, Plant Science and Biodiversity Center, 950 07 Nitra, Slovakia; veronika.lancikova@savba.sk

**Keywords:** grain amaranth, γ radiation, starch synthesis genes, droplet digital PCR

## Abstract

We investigated the expression pattern of four major starch genes at different seed developmental stages in the radiation-bred amaranth variety “Pribina” (*Amaranthus cruentus* L.) and corresponding control genotype “Ficha” (*Amaranthus cruentus* L.). Two platforms were used and compared for the gene expression analysis of *GBSSI*, *SSSI*, *SBE*, and *DBE* amaranth genes, including a standard quantitative real-time PCR (qPCR) technique and relatively novel droplet digital PCR (ddPCR) assay. In our conditions, both methods showed great accuracy and revealed higher expression of the investigated genes in the mutant variety than in the control genotype. Here we report for the first time, a ddPCR gene expression assay for the cultivated grain amaranth, as the most important group of the species in the genus *Amaranthus*.

## 1. Introduction

Gene expression analysis is among the most commonly used methods in current biology. Gene expression profiling can be done by real-time PCR, next-generation sequencing technology, microarray- or hybridization-based assays. Quantitative real-time PCR (RT-qPCR) has revolutionized the quantitative assessment of mRNA expression and to date represents the method of choice for accurate measurement and quantification of gene expression. In addition to quantification, the RT-qPCR approach can be used for copy number estimation, DNA methylation analysis, and genotyping [1,2]. However, there are potential system errors represented by biological and technical variations that influence the quantitative assessment of qPCR [3,4]. Therefore, reference genes are chosen to remove the technical variation from the final calculations and to normalize the results of qPCR expression experiments. Generally, reference genes represent stable expression levels because they are not influenced by any experimental conditions, like biotic and abiotic stress, and their expression is not specific to any developmental stage, tissue type, or organ. In this sense, the selection of a suitable internal control gene is crucial to the success of a gene expression experiment. Although only one reference gene is common practice, using a group of reference genes is a best practice that allows for more reliable quantification. There are two quantification strategies of target DNA molecules, relative and absolute quantification. Relative quantification is based on the comparison of the amount of a studied gene to the amount of a reference gene. In case of the absolute quantification standard curve must be employed during analysis, and an exact copy of the number of target DNA is compared to the DNA standards.

The recently emerged method of droplet digital PCR (ddPCR) was developed to provide precise absolute quantification of nucleic acid target sequences without the use of standard curves [5,6,7,8]. This high precision method is based on the amplification of single target DNA molecules. The key aspect of this revolutionary quantification technology is sample fragmenting into water–oil based emulsion microdroplets and PCR amplification of the target DNA molecules in each individual droplet. Each PCR sub-reaction contains either a few or no target sequences. Thereby, ddPCR may facilitate the measurement of low abundant targets [6,9,10,11]. This novel method of molecular biology is mainly used in medical research and diagnostic applications but has also been implemented in environmental science [12,13,14], plant pathogen detection and quantification [15,16,17], and food and feed safety control associated with GMO determination [18,19,20,21].

We analyzed the gene expression of four major starch amaranth enzymes during seed development in the radiation-developed variety “Pribina” (*Amaranthus cruentus* L.) and the corresponding control genotype “Ficha” (*Amaranthus cruentus* L.). Two expression quantification assays, including standard qPCR and droplet digital PCR platforms, were used.

The aim of this study was to assess the applicability of the digital PCR method for gene expression analysis to the amaranth genome and to assess which method is most suitable for the quantification of gene expression. We compared the performance of both approaches for quantitative gene expression measurement of granule-bound starch synthase (*GBSSI*) catalyzing amylose synthesis, soluble starch synthase (*SSSI*), starch branching enzymes (*SBE*), and starch debranching enzymes (*DBE*) that catalyze the synthesis of amylopectin [22,23,24,25,26].

## 2. Results and Discussion

### 2.1. Optimization of qPCR and ddPCR Assay

**Figure 1:**
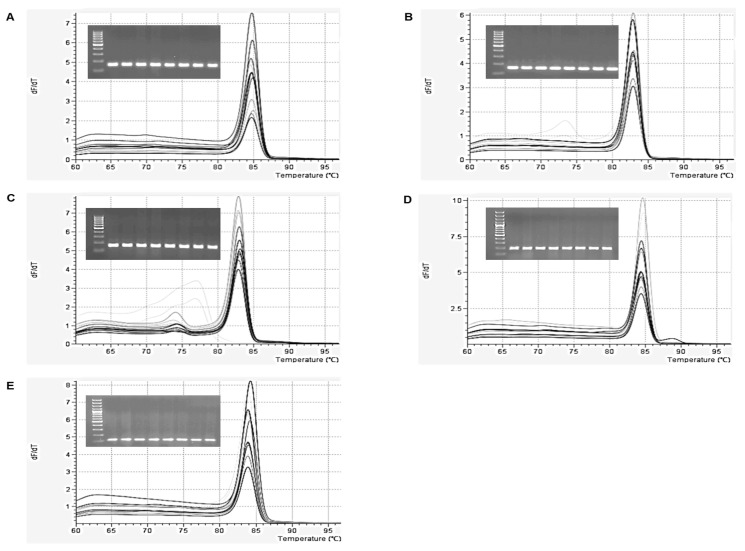
Specificity of primers and efficiency of investigated genes for the qPCR assay. Traces of melt analysis and gel electrophoresis for reference housekeeping gene *ACT* (**A**) and amaranth starch synthase genes *GBSSI* (**B**), *SSSI* (**C**), *SBE* (**D**), and *DBE* (**E**).

The qPCR assay was optimized for amaranth starch synthase genes. Primer specificity of the analyzed targets was evaluated using primer BLAST. Validation of the qPCR assay was performed using a standard curve to assess primer efficiency, linear dynamic range, and reproducibility. The primer efficiency showed good reaction efficiency (between 90–110%) and the melt curve and gel analysis confirmed the presence of desired PCR amplicons as unique and single peaks for all analyzed genes (Figure 1).

Subsequently, the qPCR assay was transferred to the ddPCR format. The same primer concentration was used for the absolute quantification assay; however, the recommended amplification ddPCR Supermix was applied. The same primers and probes were used for digital PCR as for qPCR [27]. An accurate approach, as the nature of the investigated sample and the efficiency of various polymerases in different sample backgrounds are critical [28]. Specifically, recalcitrant starch-rich amaranth seeds may contain inhibitory compounds that might cause loss of sensitivity, accuracy, or reproducibility. For ddPCR, Maheshwari et al. (2017) [29] observed reliable, reproducible results and higher tolerance to PCR inhibitors. The same conclusion was reported by Coudray-Meunier et al. (2015) [30] when analyzing Norovirus and Hepatitis A virus in samples of lettuce using microfluidic digital PCR.

**Figure 2:**
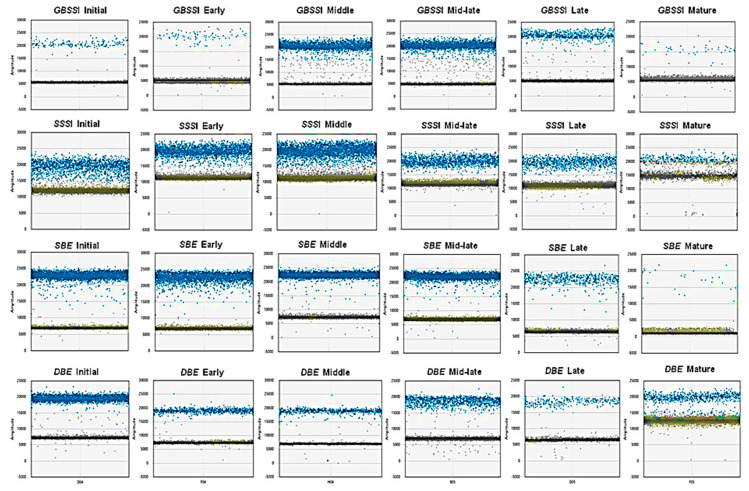
Raw data platform generated by the ddPCR quantification assay. Results of ddPCR showing positive (blue) and negative (grey) droplet counts for analyzed starch genes during the initial, early, middle, mid-late, and late stages of seed development and in mature seeds. Positive droplets with amplification and negative droplets with no amplification were distinguished based on the threshold settings in QuantaSoft.

Raw data obtained as droplet counts were evaluated using QuantaSoft software. Threshold values were set up automatically or manually, to discriminate positive droplets containing the target (above selected threshold) and negative droplets (below threshold, Figure 2). For ddPCR, careful analysis of the raw data is essential to obtain meaningful, reproducible results. The discrimination of positive and negative droplets can affect the credibility of results. Droplets can be identified as clearly positive or negative based on fluorescence intensity. Nevertheless, there are droplets called “rain”, exhibiting fluorescence between positive and negative. It is still unclear why rain occurs [18].

### 2.2. Absolute Quantification of Essential Amaranth Starch-Related Genes Using ddPCR

The ddPCR approach was employed to determine absolute quantities of four essential starch synthesis amaranth genes *GBSSI*, *SSSI*, *SBE*, and *DBE* in the γ radiation-bred variety “Pribina” during different stages of seed development (initial, early, middle, mid-late, late stage and mature seeds). The expression data from “Pribina” seeds were compared to the expression estimates obtained for non-irradiated control seeds “Ficha”. The mutant variety “Pribina” was characterized by the genetically fixed increased weight of 1000 seeds as a result of radiation [31,32], which might be associated with upregulation in seed starch synthesis/accumulation since starch is the most abundant component in the amaranth seed. It is reported that seed weight can be positively correlated with the starch-synthase enzyme activity in rice, maize, and wheat [33,34,35].

The transcription pattern of the investigated genes has already been described during amaranth seed development using the standard qPCR approach [22,23,26,36]. Based on the results of these previous reports, all starch synthesis genes are expressed in the storage tissues as well as in the non-storage tissues.

**Figure 3:**
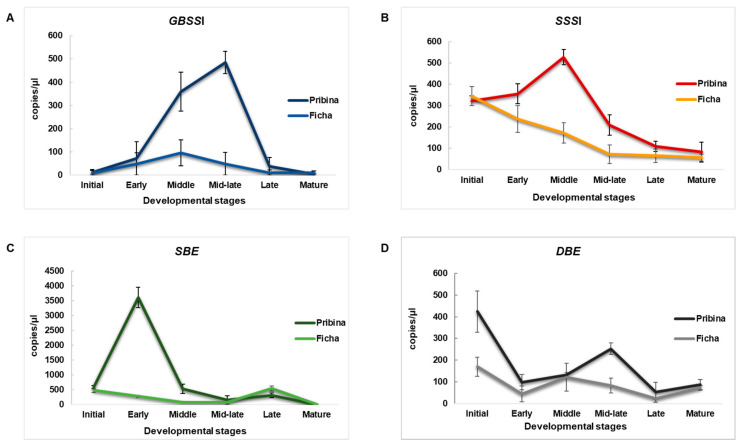
Absolute quantification pattern of amaranth starch key enzymes *GBSSI*, *SSSI*, *SBE*, and *DBE* in the mutant variety “Pribina” and control genotype “Ficha” during seed formation. The ddPCR approach was applied to determine the transcript copy number (displayed as copies/µL) of the granule bound starch synthase I (**A**), soluble starch synthase I (**B**), starch branching enzyme (**C**), and starch debranching enzyme (**D**) at different stages of seed development in the radiation-bred variety “Pribina” and control genotype “Ficha”.

In our experiment, the control genotype “Ficha” was characterized by lower gene expression of all four investigated genes whilst “Pribina” showed, as hypothesized, higher gene expression predominantly in the middle and mid-late seed developmental stages (Figure 3).

The expression peak of *GBSSI*, known as the waxy gene that catalyzes amylase synthesis, was defined to be at the mid-late developmental stage (Figure 3A). As for *GBSSI* expression in seeds, our observations were similar to those reported by Park et al. (2011) [22], according to whom the *GBSSI* was characterized as a “late expresser”, showing the transcription peak at the mid-late developmental stage. Such mRNA distribution and activity were also found in cereal endosperm [37,38]. When comparing *GBSSI* gene expression in the mutant variety and the control genotype, the mutant variety “Pribina” showed significantly higher upregulation of the *GBSSI* gene at the middle and mid-late seed developmental stages over the corresponding control “Ficha” (Figure 3A). The *GBSSI* gene encodes amylose synthesis. James et al. (2003) [39] described the high level of proportionality between enzymatic activity and *GBSSI* gene expression in cereals, however no correlation between *GBSSI* gene expression and amylose content was revealed [40,41]. Thus, amylose content may be responsive to some other factors besides *GBSSI*. Our results of starch (not published herein) suggest a similar statement.

“Ficha” seeds showed the highest gene expression of *SSSI* at the initial stage, but it slowly decreased through the mid-late stage and remained steady until seed maturation. “Pribina” seeds produced different trends in *SSSI* expression with the highest activity during the middle stage of seed formation (Figure 3B). Hence, ddPCR demonstrated the most balanced gene expression with threefold upregulation in “Pribina” seeds at the middle and mid-late stage when compared to the control “Ficha”.

The most significant difference between the two investigated amaranths was determined for *SBE* activity. “Ficha” showed relatively low transcript levels across all seed developmental stages with very limited activity at the middle and mid-late stages. In contrast, “Pribina” was characterized by a rapid increase in *SBE* activity at the initial developmental stage, which immediately decreased at the middle stage of seed development (Figure 3C).

The herein observed *SSSI* and *SBE* transcription patterns were comparable to the results reported by Park and Nishikawa (2012) [23]. These authors found the expression profiles of *SSSI* and *SBE* genes to be similar throughout seed formation. Moreover, both genes were active at relatively high levels at the initial stage of seed development with activity graduated at the middle stage and decreasing thereafter. The importance of *SBE* in amylopectin synthesis has been previously discussed. The starch branching enzyme plays a fundamental part in amylopectin synthesis, catalyzes the formation of *α*-1, 6-linkages, and decides the branching pattern in amylopectin [42]. Wang et al. (2017) [43] concluded that the suppression of *SBE* had a crucial impact on obtaining of high-amylose lines of maize and rice.

The pattern of expression profiles of the *DBE* enzyme showed the highest similarity in the two analyzed amaranths with activity mainly restricted to the initial, followed by middle and mid-late stages of seed formation (Figure 3D). Park et al. (2014) [26] found a rapid increase of the *DBE* transcript level at the middle stage of seed development, indicating the important role of this enzyme in the accumulation of starch throughout the seed during the middle stage of its development. However, according to the research by Park et al. (2014) [26], this gene showed weak activity during the initial stage of seed formation. Thus, the authors suggested the function of *DBE* in pericarp amylopectin synthesis at the initial stage of seed development. Similar results were reported for the rice *DBEI* transcript [44].

### 2.3. Performance of ddPCR vs. qPCR

The conventional standard curve of qPCR provides the fold change of target genes in experimental samples relative to control samples, while ddPCR determines absolute gene expression without the need for a standard curve, calibrator, or reference gene. Determination of the absolute copy number of targeted genes is essential in many cases, but the success of absolute quantification relies on an accurate standard.

Only a limited number of studies directly compare the performance of the golden standard qPCR vs. third-generation digital PCR. Some studies reported improvement of target DNA detection sensitivity using the ddPCR approach over the qPCR method [15,45,46]. As for other studies, similar or the same limit of detection for both quantification methods was reported [15,47]. However, digital PCR might be beneficial in the detection of low abundant transcripts with small expression differences [29] and offers a relatively simple and straightforward tool for the absolute quantification of transgenes [47].

To compare the standard qPCR technique with the innovative ddPCR quantification method, identical cDNA samples were used to simultaneously set up these experiments.

**Figure 4:**
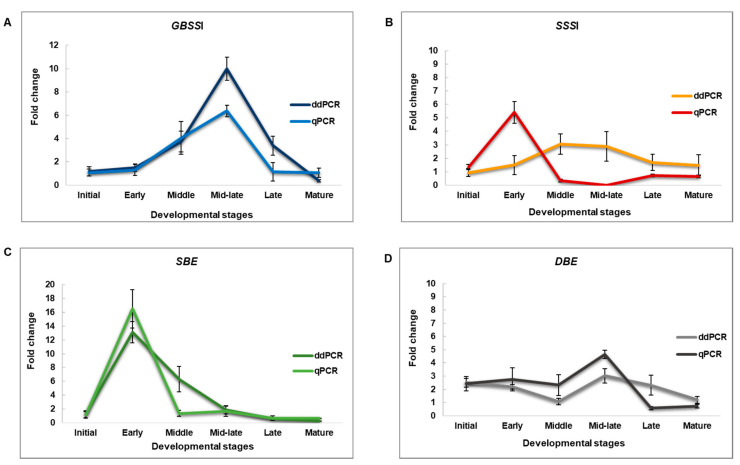
Comparison of ddPCR and qPCR performance. Fold change in gene expression of the granule bound starch synthase I (**A**), soluble starch synthase I (**B**), starch branching enzyme (**C**), and starch debranching enzyme (**D**) during seed development of investigated amaranths. Fold change for ddPCR was estimated as a ratio of “Pribina” and “Ficha” absolute transcript quantities, the conventional standard qPCR curve was normalized against reference gene *ACT*.

Both approaches showed upregulation of *GBSSI* expression in the mutant variety “Pribina” at the middle and mid-late stages of seed development compared to the control genotype “Ficha” (4A). Approximately fourfold *GBSSI* upregulation was observed by both the quantitative PCR methods at the middle stage of seed formation. The qPCR approach revealed a sixfold increase in *GBSSI* expression at the mid-late seed developmental stage, whilst up to a tenfold change in expression was observed using ddPCR. The downward trend in gene expression was revealed at the late stage of seed development with a complete decrease of the transcript copy number in mature seeds (Figure 4A). These divergences in the *GBSSI* gene activity trend obtained by relative qPCR and absolute ddPCR may be caused by a low abundance of the *GBSSI* transcript. In cases where it is necessary to measure low copy transcripts in a given sample, ddPCR might be a more accurate and sensitive approach. Furthermore, a lack of sufficient details on reference genes in amaranth, predominantly during seed development, might lead to variability in the expression of key enzymes that catalyze amylose synthesis. Recently, Vera Hernandéz et al. (2018) [48] analyzed seven housekeeping genes from *Amaranthus hypochondriacus* using qPCR in different tissues, developmental stages, and under different stress conditions. *AhyMDH*, *AhyGAPDH*, *AhyEF-1α*, and *AhyACT* were evaluated as the most stable and suitable genes for data normalization. However, the stability of *Actin* during seed development was not discussed. Therefore, the variability of *Actin* as a reference gene in developing seeds cannot be ruled out.

Soluble starch synthase I, encoding amylopectin synthesis, was the only one of the four investigated starch genes where ddPCR and qPCR produced inconsistent results (Figure 4B). The qPCR method showed rapid gene upregulation at the early stages of seed development, an immediate decrease following the seed formation stage, and remained constantly expressed until the end of seed development. Taking into account the relatively low transcript abundance of the *SSSI* gene and *GBSSI* gene, ddPCR can be considered a more precise quantification method as it eliminates the effect of reference genes.

Both approaches yielded consistent results for the starch branching enzyme *SBE* (Figure 4C). The most abundant *SBE* transcript was observed at the early stage of seed development, where the ddPCR and qPCR platforms showed thirteenfold and nearly seventeenfold upregulation, respectively of *SBE* in “Pribina”.

The starch debranching enzyme *DBE* showed the most comparable trend in gene expression across all analyzed seed developmental stages (Figure 4D). A similar level of modest upregulation at the mid-late seed stage was shown by both tested quantification methods.

In this work, four starch-related genes showed a certain level of upregulation in the mutant variety “Pribina” characterized by higher quantitative seed traits (weight, size) over the control “Ficha”. *GBSSI*, *SSSI*, and *DBE* genes were moderately elevated. However, the most significant upregulation was observed in the *SBE* gene, one of the genes encoding the synthesis of amylopectin. Our results support suggestions that the improvement of quantitative seed attributes may be associated with increased starch-synthase enzyme activity or starch content. Moreover, our results show the applicability of the dPCR approach in the study of gene expression analysis in grain amaranth (*Amaranthus cruentus* L.).

## 3. Materials and Methods

### 3.1. Plant Material and Experimental Field

Gamma irradiation mutagenesis was previously applied to the seeds of genotype “Ficha” (*A. cruentus*), and several mutant lines with improved quantitative and qualitative seed traits were selected [31,49,50]. Two selected mutants were registered as new varieties [32]. The radiation mutant variety “Pribina” (*A. cruentus*), characterized by a high weight of 1000 seeds and increased seed size, and its non-irradiated counterpart “Ficha“ (*A. cruentus*) were used for this study.

Amaranth plants genotype “Ficha“ and variety “Pribina” were cultivated on the experimental field at the locality Nitra, 290 m above sea level, with annual precipitation of 600 mm and mean annual temperature of 9.5 °C. The cultural practices have been reported in our previous work [32].

The seeds were sown at the beginning of May 2018 and collected for gene expression analysis in different developmental stages according to Park et al. 2011 [22] (initial, early, middle, mid-late, late, and mature seeds). Mature seeds were collected for analysis at the end of September 2018. All seed samples used in this study were harvested at each developmental stage in three independent biological replicates.

### 3.2. Gene-Specific Primer Design

Gene-specific primers of four genes of major importance in amaranth starch biosynthesis were designed based on the nucleotide sequence of *Amaranthus cruentus* L.

The genes were amplified using the following primer pairs: granule bound starch synthase I (*GBSSI*, GenBank AB456685) forward 5′-ATGGAAACAGTAACATCTTCTCACT-3′ and reverse 3′-GTACTTTTTGGGTTGTTGCTTAATCT-5; soluble starch synthase I (*SSSI*, GenBank AB626804) forward 5′-AGTGAGAACCTACAGGGATTACAAGG-3′, and reverse 5′-GTATGGAGGATCAATGAGTGCCCATT-3; starch branching enzyme (*SBE*, GenBank AB872446.1) forward 5′-AGATCTGGAAACCCCGAGGA-3′, reverse 3′-AGGATTCCTGTGCACCTTGG-5′; starch debranching enzyme (*DBE*, GenBank AB822998.1) forward 5′-GGTGAGTTAGCACCTGAAGATAG-3′, reverse 3′-CTTTGTGGGAGCTTAAGAGGAA-5′.

Droplet digital PCR and quantitative PCR were performed using the same primer set for each starch gene. For the relative gene expression analysis using standard qPCR, the reference gene *Actin* from *Amaranthus tricolor* (*ACT*, GenBank EF452618) was applied (Park et al. (2012) using the forward 5′-GTATGCAAGTGGTCGTACTACAGG-3′, and reverse 3′-ATCTTCGTAGGGTAATCAGTCAGG-5′ primer pair.

### 3.3. RNA Extraction and cDNA Synthesis

Seed material was ground into a fine powder using the TissueLyser II (Qiagen, Hilden, Germany). Total RNA was extracted according to the protocol developed for cereal seeds containing a high level of starch [51] from approximately 0.2 g of seed powder. RNA quality and quantity were measured using a Nanodrop spectrophotometer and RNA integrity was verified on a 1.5% agarose gel. The cDNA was synthesized from 300 ng RNA using a Maxima First Strand cDNA Synthesis Kit for RT-qPCR, with dsDNase treatment (ThermoFisher Scientific, Waltham, MA, USA). To minimize variability between ddPCR and qPCR, the same cDNA samples were used for both approaches.

### 3.4. Relative Gene Expression Analysis Using qRT-PCR

The qRT-PCR method was carried out using a LightCycler^®^ Nano (Roche, Basel, Switzerland). The gene-specific primers were evaluated. The primer pair specificity was evaluated using primer BLAST. The qPCR assay validation was conducted using a standard curve to assess primer efficiency, linear dynamic range, and reproducibility. The reaction mixture consisted of 2× Sso Advanced Universal SYBR^®^ Green supermix (BioRad, Hercules, CA, USA), 400 nM of each forward and reverse primer, 1.5 µL of diluted cDNA, and nuclease-free water added up to the total reaction volume of 10 µL.

The three-step thermal cycling protocol for *GBSSI* was applied as follows: 95 °C for 30 s, 45 cycles of denaturation at 95 °C for 15 s, annealing at 60 °C for 45 s, and elongation at 72 °C for 30 s. The following two-step thermal cycling protocol was applied for *SSSI*, *SBE* and *DBE*: 95 °C for 30 s, 45 cycles of denaturation at 95 °C for 15 s, and annealing/elongation at 60 °C for 1 min. Melting curves were generated for each analyzed gene after a qPCR run. Target samples were normalized against *Actin* and data were analyzed using the LightCycler Nano software.

### 3.5. Absolute Gene Expression Analysis Using ddPCR

The same primer concentration was used for the ddPCR assay as for qRT-PCR. The reaction mixture for the ddPCR assay consisted of 2× QX200^TM^ ddPCR^TM^ EvaGreen Supermix (Bio-Rad, Hercules, CA, USA), 400 nM of each forward and reverse primer, 1.5 µL of diluted cDNA, and molecular grade water added up to total reaction volume of 20 µL.

A disposable eight-channel DG8 cartridge was placed into the cartridge holder, then the total volume of the PCR mixture was transferred into the middle wells of the cartridge and the bottom wells were filled with 70 µL of droplet generation oil (QX200^TM^ Droplet Generation Oil for EvaGreen, Bio-Rad). The cartridge containing the PCR reaction mixture and droplet generation oil was placed into the QX200^TM^ Droplet Generator (Bio-Rad, Hercules, CA, USA). The 40 µL of final emulsion containing droplets were transferred from the DG8 cartridge into the ddPCR^TM^ 96-well PCR plate (Bio-Rad). The PCR plate was heat-sealed at 175 °C for 3 s with pierceable foil using a PX1 PCR plate sealer (Bio-Rad, Hercules, CA, USA). Subsequently, PCR amplification was carried out in a C1000 Touch^TM^ Thermal Cycler with a 96-Deep Well Reaction Module (Bio-Rad) and consisted of initial denaturation at 95 °C for 5 min followed by 45 cycles of denaturation at 95 °C for 30 s and annealing/elongation at 60 °C for 1 min with a ramp of 2 °C/s. Final signal stabilization was performed at 4 °C for 5 min followed by 95 °C for 5 min. Then, the PCR plate containing the droplets was placed in a QX200^TM^ droplet reader (Bio-Rad, Hercules, CA, USA) to count positive and negative droplets. Data were analyzed using QuantaSoft^TM^ Software.
